# Efficacy of Composite Restorative Techniques in Marginal Sealing of Extended Class V Cavities

**DOI:** 10.5402/2011/180197

**Published:** 2010-10-03

**Authors:** Salwa Khier, Khamis Hassan

**Affiliations:** ^1^Dental Biomaterials, Restorative Dental Sciences Department, College of Dentistry, King Saud University, Riyadh 11545, Saudi Arabia; ^2^Operative Dentistry, Restorative Dental Sciences Department, College of Dentistry, King Saud University, P.O. Box 60169, Riyadh 11545, Saudi Arabia

## Abstract

*Objectives*. To compare the efficacy of three placement techniques in marginal sealing of Class V composite restorations extending onto the root. *Materials and Methods*. Class V cavities were prepared on buccal surfaces of 30 extracted human molars, with gingival margins 1.5 mm on the root. Prepared teeth were randomly assigned into 3 groups of 10 each and restored with Single Bond/Filtek Supreme using following techniques; Group I: oblique; Group II: occlusogingival; and Group III: split-increment. After restoration finishing, teeth were thermocycled, and immersed in 2% methylene blue dye for 24 h. Teeth were sectioned buccolingually. Digital photographs were made of sectioned surfaces using digital camera fitted on stereomicroscope. Microleakage was scored at occlusal and gingival margins using 0–3 scale. Dye penetration depth (DPD) at both margins was also measured using AnalySIS software. Data were analyzed using one-way ANOVA and Bonferroni multiple comparison test. *Results*. 5% of occlusal margins in Groups I and III had 50 *μ*m average (DPD). In Group II, only 10% of occlusal margins showed dye penetration, with 60 *μ*m average depth. For gingival margins, Groups I and III presented dye penetration in 55% of specimens, with 220 and 150 *μ*m average (DPD), respectively. Group II had 60% of gingival margins, with 230 *μ*m average (DPD). There was no significant difference in microleakage at occlusal and gingival margins in all groups. Dye penetration was larger at gingival than at occlusal margins (*P* < .001). *Conclusion*. None of placement techniques produced gap-free margins. Oblique and occlusogingival techniques exhibited higher degrees of microleakage at occlusal and gingival margins, as compared to that of split-increment technique. Splitting flat composite increment by diagonal cut, prior to light-curing, preserved bonded gingival margin integrity and reduced microleakage.

## 1. Introduction

Composite resins possess the inherent problem of shrinkage during light polymerization which generates stress [1, 2]. One of the major challenges in restorative dentistry is to control this stress and achieve a direct composite restoration with complete and long-lasting marginal seal. Despite their direct access, cervical cavities restored with composite resins pose an additional challenge, especially when extending beyond the cementoenamel junction because of variation in occlusal and gingival hard tooth structure [3, 4]. 

The shrinkage stress generated within the bonded composite restoration is transferred to the adhesive interface between the cavity walls and the composite resin [1, 2, 5]. This stress pulls away the shrinking composite resin from the cavity walls and results in breaking the weaker adhesive bond with gingival dentin, resulting in marginal gap formation [6–8]. The marginal gap leads to penetration of bacterial fluids, molecules, and ions into the margins of restorations [9, 10] and results in postoperative sensitivity, discoloration, recurrent caries, and pulpal complications [11–13]. 

The effect of the shrinkage stress on the marginal gap formation depends on the magnitude of such stress in relation to the interfacial bond strength [7]. The magnitude of this stress is influenced by several factors including the type of composite resin, modulus of resin elasticity, C-factor, restorative techniques, polymerization rate, and polymerization technique [14–16].

Among the efforts made to minimize the extent of interfacial gaps in Class V composite restorations is the use of incremental restorative techniques [17, 18]. Several such techniques [19–25] were proposed and used, including the recently introduced split-increment technique [26], with the objective of minimizing the consequences of polymerization shrinkage and achieving a better marginal adaptation [27–29]. 

Several research studies evaluated the effect of placement techniques on microleakage of Class V composite restorations [8–10, 29–31]. 

The aim of this in vitro study was to qualitatively and quantitatively assess and compare microleakage of Class V composite restorations at occlusal and gingival margins extending onto the root, that were placed using three different restorative techniques.

## 2. Materials and Methods

### 2.1. Specimen Preparations

Thirty sound, freshly extracted human molars were selected to be used in this study and were stored in physiologic solution at room temperature. Standardized Class V cavities (4 mm wide, ×  3 mm long, ×  2 mm deep) were prepared on buccal surfaces where gingival margins were located 1.5 mm in cementum/dentin and occlusal enamel margins were beveled. All cavity preparations were done by a single calibrated operator using no. 245 carbide burs (SS White, Great White Series, Lakewod, NJ, USA) at a high-speed handpiece under water cooling. Four cavities were cut using one bur to avoid dullness.

### 2.2. Restorative Procedures

The prepared teeth were randomly divided into three groups of 10 each. The same calibrated operator performed all restorative procedures. In all groups, the total-etch technique was performed prior to the adhesive application. A 35% phosphoric acid (Scotchbond Etchant, 3M-ESPE, St. Paul, MN, USA) was applied initially to beveled enamel margins and then extended to the cavity floor for 15 seconds. The acid was rinsed with air/water spray for 15 seconds, and excess moisture was removed with a minisponge applied on dentin while enamel was gently air-dried. Single Bond adhesive system (3M ESPE, St. Paul, MN, USA) was applied according to the manufacturer's instructions to preparations in all groups and was light-cured for 10 seconds. All cavities were restored with Shade A2-Filtek Supreme nanofilled composite resin (3MESPE, St. Paul, MN, USA). Each increment was light-cured for 20 seconds using Elipar Trilight, quartz-tungsten halogen light (3M ESPE, St. Paul, MN, USA). The light-curing unit was calibrated at 500 mW/cm^2^ and the curing tip was constantly positioned at a distance of 0.5 mm from the surface of each restoration. 

The prepared cavities in each experimental group were restored using one of the three composite restorative techniques. In Group I, the oblique incremental technique [4] was utilized where the occlusal wedge-shaped increment was positioned first to fill half of the cavity, adapting to the entire occlusal and axial walls, and then light-cured. The second gingival increment filled the rest of the cavity and was then light-cured. In Group II, the occlusogingival incremental technique [24] was used. The occlusal increment was placed first to fill the occlusal half of the cavity and was light cured. The second gingival increment filled the rest of the cavity sealing the gingival dentin margin and was light cured. In Group III, the split-increment technique [26] was used where a 2 mm thick flat composite resin increment was applied to fill the cavity. Prior to light-curing, a diagonal cut was made in this increment using a blunt plastic filling instrument in a push stroke. This cut was 1.5 mm wide and extended the entire thickness of the resin increment, splitting it into two triangular-shaped flat portions. Composite resin was then light-cured. Following light-curing, the diagonal cut was filled with composite resin and light-cured. All restorations were immediately finished, following a standard finishing method, with Sof Lex Pop-On discs (3M ESPE, St. Paul, MN, USA).

### 2.3. Assessment Procedure

The restored teeth were stored in distilled water 37°C for 24 hours. The restored teeth were subjected to thermal cycling of 600 cycles in a 5°–55°C water bath with a dwell time of 60 seconds in each bath. After that the teeth were retrieved and their apices sealed with Cavit-G (ESPE, Norristown, PA, USA). All tooth surfaces were covered with two coats of fingernail polish, with the exception of 1 mm around the tooth-restoration interface. The teeth were then immersed in a 2% methylene blue dye solution at room temperature for 24 hours. Teeth were removed, rinsed with tap water, and air-dried. Fingernail polish was scrapped off, and teeth were embedded in self-curing transparent acrylic resin. 

Teeth were sectioned longitudinally in buccolingual direction through the center of the restoration with a diamond saw (Isomet, Buehler Ltd, Lake Buff, IL, USA) at low speed under water, yielding two specimens per tooth (20 specimens per group). Digital photographs were made of sectioned surfaces of all specimens using Nikon digital camera DXM1200 fitted on stereo microscope (Leica MZ16 FA, Switzerland) at 30x magnification. Specimens of the six technique/margin combinations were evaluated at the occlusal and gingival margins for the extent of dye penetration. Specimens exhibiting dye penetration ≥0.1 mm beyond the cavosurface margin were considered to have microleakage. Dye penetration was examined by two independent evaluators precalibrated at 85% reliability. If any disagreements in score between the two evaluators were reported, the higher score was taken. The extent of dye penetration was scored as follows: 0 : no  dye  penetration; 1 : dye  penetration up to 1/3 along the occlusal/gingival wall; 2 : dye  penetration up to 2/3 along the occlusal/gingival wall without reaching the axial wall; 3 : dye  penetration reaching the axial wall. The depth of dye penetration was also measured (microns) at the occlusal and gingival margins of each specimen using AnalySIS (Soft Imaging System) software. Data were analyzed using one-way ANOVA test with a Bonferroni correction for pairwise multiple comparisons at a significance level of *P* < .05.

## 3. Results

The mean microleakage score and depth of dye penetration at occlusal and gingival margins in all groups are presented in Tables 1 and 2, respectively. None of the restorative placement techniques tested in this study completely eliminated microleakage. 5% of the occlusal margins in Groups I and III had an average depth of dye penetration of 50 *μ*m. In Group II, only 10% of the occlusal margins showed dye penetration, with an average depth of 60 *μ*m. For gingival margins, Groups I and III presented dye penetration in 55% of the specimens, with an average dye penetration depth of 220 and 150 *μ*m, respectively. Group II had 60% of the gingival margins with dye penetration (average depth of 230 *μ*m).

The summary of statistical analysis of the data obtained for the depth of dye penetrations are presented in Table 3. There was no significant difference in microleakage at the occlusal and gingival margins in all tested groups. Dye penetration was larger at gingival margins than at occlusal margins (P < .001). Bonferroni multiple comparison test at 95% confidence revealed no statistically significant difference in microleakage between the occlusal and gingival margins (*P* = .127).

## 4. Discussion

Several microleakage studies [8–10, 19–21, 25, 29–31] were conducted to assess the effect of composite placement techniques on microleakage of Class V composite restorations. Some of which demonstrated that the incremental techniques result in an improved marginal sealing [19–21, 25]. 

In the present study, microleakage was not completely eliminated by the investigated restorative placement techniques. However, these techniques showed a decrease in microleakage with a lower degree recorded at the occlusal margin compared to that at the gingival margin. The lower degree of microleakage exhibited by all occlusal margins in the present study was not surprising and can readily be explained by the presence of enamel at these walls and margins which provided a stronger adhesive bond that has overcome the weaker dentin bond at the gingival wall. The reduction in microleakage observed in the present study is consistent with the findings of previous studies [19–21, 25]. 

The oblique incremental and the occlusogingival incremental techniques showed higher degrees of microleakage at both the occlusal and gingival margins; however, this increased microleakage was not significantly different from that recorded for the split-increment technique. 

The lower degree of microleakage exhibited by the split-increment technique in this study could be related to the diagonal cut made in the composite increment prior to application of the curing light. This precuring diagonal cutting of the flat increment has led to bonded occlusal and gingival opposing cavity walls not being connected by a single composite increment. This increment splitting resulted in improving polymerization shrinkage stress developed at the bonded cavity walls and margins by converting the restricted shrinkage to unrestricted shrinkage [14, 15]. The increment splitting prevented the pulling ability of the strong enamel bond at the occlusal wall against the weak dentin bond at the gingival wall [7], with subsequent preservation of the gingival margin integrity and reduction of microleakage. 

The diagonal cutting of the flat composite increment into two triangular composite portions has created new unbonded composite surfaces which served as additional reservoirs for flow or plastic deformation during light polymerization [14, 15], and eventually preserved the interfacial bond and marginal integrity of the restoration. Moreover, this diagonal splitting of the flat composite increment has resulted in reduction of the C-factor from 5.0 to almost 0.7. The decreased C-factor resulted in minimizing the polymerization shrinkage stress formed within the composite restoration and consequently reduced its destructive effect on the adhesive interfaces and cavity margins [1, 2]. 

The actual number of composite increments needed to restore a cavity, in general, depends on the volume of space undergoing restoration, with larger lesions requiring more incremental applications. As the prepared cavity, in the present study, has a depth of 2 mm, a single flat composite increment was used and provided with a single diagonal cut prior to light curing. 

The split-increment technique could be useful for restoring large Class V cavities with composite resins, especially when gingival margins extend beyond the cement-enamel junction. Such restorations would preserve the gingival marginal seal and reduce microleakage.

## 5. Conclusion

None of the composite restorative techniques used in this study for restoring extended Class V cavities produced gap-free margins. The oblique incremental and the occluso-gingival incremental techniques exhibited higher degrees of microleakage at the occlusal and gingival margins, as compared to those of the split-increment technique. 

The splitting of composite flat increment, by a diagonal cut, prior to light curing preserved the bonded gingival margin integrity and reduced microleakage.

## Figures and Tables

**Table 1:** Microleakage score and depth of dye penetration at occlusal margin.

Group	Scores				Depth of dye penetration (*μ*m)
	0	1	2	3	
I	19	1	0	0	50
II	18	2	0	0	60
III	19	1	0	0	50

**Table 2:** Microleakage score and depth of dye penetration at gingival margin.

Group	Scores				Depth of dye penetration (*μ*m)
	0	1	2	3	
I	9	11	0	0	220
II	7	12	1	0	230
III	9	11	0	0	150

**Table 3:** Statistical analysis summary of depth of dye penetration (*μm*) at the occlusal and gingival margins.

Technique (group)	Depth of dye penetration (*μ*m)		One-way ANOVA	Bonferroni-*t* Test		
	Margin	Mean	*P*-value	Group/margin	Group/margin	*P*-value
I	Occlusal	50	.056	I/Occlusal	II/Occlusal	.086
	Gingival	220		I/Gingival	II/Gingival	.067
II	Occlusal	60	.127	II/Occlusal	III/Occlusal	.075
	Gingival	230		II/Gingival	III/Gingival	.076
III	Occlusal	50	.072	III/Occlusal	I/Occlusal	.064
	Gingival	150		III/Gingival	I/Gingival	.075

The mean difference is significant at *P* < .05.
